# Systematic evaluation of protein extraction for metaproteomic analysis of marine sediment with high clay content

**DOI:** 10.1093/ismeco/ycaf074

**Published:** 2025-05-22

**Authors:** Anne Ostrzinski, Benoit J Kunath, André Rodrigues Soares, Cédric C Laczny, Rashi Halder, Jens Kallmeyer, Rolando di Primio, Paul Wilmes, Alexander J Probst, Anke Trautwein-Schult, Dörte Becher

**Affiliations:** Department of Microbial Proteomics, Institute of Microbiology, University of Greifswald, 17489 Greifswald, Germany; Luxembourg Centre for Systems Biomedicine, University of Luxembourg, 4362 Esch-sur-Alzette, Luxembourg; Department of Environmental Metagenomics, Research Center One Health Ruhr, University Alliance Ruhr, Faculty of Chemistry, University of Duisburg-Essen, 45141 Essen, Germany; Luxembourg Centre for Systems Biomedicine, University of Luxembourg, 4362 Esch-sur-Alzette, Luxembourg; Luxembourg Centre for Systems Biomedicine, University of Luxembourg, 4362 Esch-sur-Alzette, Luxembourg; Subsurface Geochemistry, Section Geomicrobiology, GFZ Helmholtz Centre for Geoscience, 14473 Potsdam, Germany; Exploration Manager Play Analysis and Access, Aker BP, 1366 Lysaker, Norway; Luxembourg Centre for Systems Biomedicine, University of Luxembourg, 4362 Esch-sur-Alzette, Luxembourg; Department of Life Sciences and Medicine, Faculty of Science, Technology and Medicine, University of Luxembourg, 4362 Esch-sur-Alzette, Luxembourg; Department of Environmental Metagenomics, Research Center One Health Ruhr, University Alliance Ruhr, Faculty of Chemistry, University of Duisburg-Essen, 45141 Essen, Germany; Department of Microbial Proteomics, Institute of Microbiology, University of Greifswald, 17489 Greifswald, Germany; Department of Microbial Proteomics, Institute of Microbiology, University of Greifswald, 17489 Greifswald, Germany

**Keywords:** Metaproteomics, marine sediment, protein extraction

## Abstract

Marine sediments harbor extremely diverse microbial communities that contribute to global biodiversity and play an essential role in the functioning of ecosystems. However, the metaproteome of marine sediments is still poorly understood. The extraction of proteins from environmental samples is still a challenge, especially from marine sediments, due to the complexity of the matrix. Therefore, methods for protein extraction from marine sediments need to be improved. To develop an effective workflow for protein extraction for clayey sediments, we compared, combined and enhanced different protein extraction methods. The workflow presented here includes blocking of protein binding sites on sediment particles with high concentrations of amino acids, effective cell lysis by ultrasonic capture, electro-elution, and simultaneous fractionation of proteins. To test the protocol’s efficacy, we added *Escherichia coli* cells to sediment samples before protein extraction. By using our refined workflow, we were able to identify a comparable number of *E. coli* proteins from the supplemented sediment to those from pure *E. coli* cultures. This new protocol will enable future studies to identify active players in clay-rich marine sediments and accurately determine functional biodiversity based on their respective protein complements.

## Introduction

Marine sediments harbor as many microorganisms as the overlaying seawater and exhibit similar levels of microbial richness, although the amount and diversity of microorganisms decreases with increasing depth and age of the sediment [1]. Therefore, studying respective microbiomes can provide insights into processes such as the biogeochemical cycling of elements, organic matter decomposition and remineralization [1], as well as information about the microbial responses to environmental changes [2]. In times of multi-omic technologies, environmental samples can be analyzed on various levels: Firstly, 16S ribosomal ribonucleic acid sequencing and metagenomics can be used to determine microbial richness and diversity in environmental samples. Secondly, metatranscriptomics provides insights into the active metabolism of present microbes, but messenger ribonucleic acid levels do not necessarily reflect the actual metabolic activity [2]. Thirdly, metaproteomics provides information about the actual level of proteins as metabolic products and, therefore, the function of microbes in a community [3].

While numerous studies have been conducted investigating marine sediments on a genomic level (1008 results found on NCBI/PubMed when searching for “metagenomics marine sediment” as of 20 July 2024), only few studies have focused on metaproteomics in these sample types (27 results for “metaproteomics marine sediment”). Several reviews on metaproteomics in marine environments have been published [4–6]. However, the study of sediments is associated with several challenges. Additional to the limited cell amount, and therefore low protein content, the high microbial diversity complicates protein identification after mass spectrometric measurements. To overcome this challenge, pre-fractioning of proteins [7, 8] and sample-specific metagenome data are required for efficient protein identification [8, 9], although numerous proteins found in marine sediments remain uncharacterized and their functions are still unknown [6]. Furthermore, efficient protein extraction also presents a considerable challenge for metaproteomic studies from marine sediments due to the high concentrations in clay minerals and organic matter such as humic substances [10]. Humic substances such as polyphenolic compounds are co-extracted with the proteins and therefore interfere with colorimetric assays for protein quantification [11, 12]. Additionally, they can modify proteins and directly impede peptide and protein detection and identification [13]. Clay concentrations can vary strongly between different marine sediments depending on the depositional environment [14]. High clay concentrations in the sediment can lead to the adsorption of proteins on sediment particles [15, 16] which massively reduce the efficiency of protein extraction [10, 17]. However, blocking potential binding sites on sediment particles with amino acids can reduce the protein binding to the material and improve protein recovery.

In the context of interfering substances, many studies to date have focused on protein extractions from soils, which may also contain high concentrations of humic substances and clay [18–29]. Protein extraction is generally influenced by soil type, with fewer proteins extracted from soils with a higher clay content [22]. Only very few studies have directly compared protocols for protein extraction from marine sediments [30–32]. Most extraction protocols include a centrifugation step to separate the proteins from the sediment after cell disruption [30, 32, 33]. Moore et al. used an electric field to pull the charged proteins directly into an SDS-gel from the sediment-buffer slurry [31, 34]. However, these studies did not focus on sediments with particularly high clay content.

The aim of this study was to develop an efficient protein extraction protocol for marine sediments with high clay content. The efficiency of protein extraction was measured using the recovery of supplemented *Escherichia coli* proteins. Pretreatment with amino acids was tested as well as different methods of cell disruption, protein extraction and purification. The final extraction approach, which involves the addition of high concentrations of amino acids to block binding sites on sediment particles and electro-elution of proteins, enabled the identification of a comparable number of *E. coli* proteins and peptides from supplemented sediment as from pure *E. coli* cultures.

## Materials and methods

### Sediment sampling

Sediment cores were collected from the Barents Sea at water depths of approximately 350 m using a gravity corer. The cores were 1–2 m in length, and only the lowermost 20 cm were utilized for this study. The sediment has a clay mineral content of up to 60%. Samples were packed under controlled aseptic conditions and frozen at −80°C. Samples were homogenized by mixing and subsequently used for protein extraction.

### Deoxyribonucleic acid extraction and metagenome creation

The detailed workflow for DNA extraction and metagenome creation is explained in the supporting information.

### *Escherichia coli* cultivation

*E. coli* K12 was cultivated in 100 ml LB medium at 35°C in a water bath shaking at 220 rpm for about 16 h. The cell suspension was centrifuged (10 000 × g, 4°C, 10 min), the supernatant was discarded, and the remaining cell pellets were washed twice with TE buffer (50 mM Tris–HCl, pH 7.5). Cell pellets were suspended in TE buffer and cell counts were estimated based on the optical density of the culture [35].

### Optimizing protein extraction

**Figure 1:**
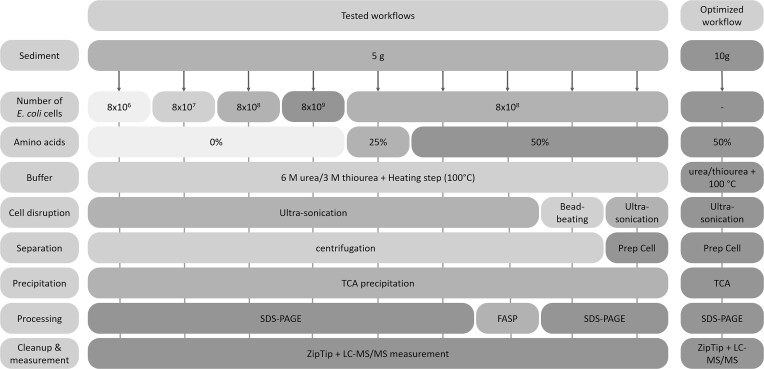
Overview of tested protein extraction steps and optimized extraction workflow. While testing the extraction workflows, all extractions were carried out in triplicates using 5 g of sediment (wet weight). Different numbers of *E. coli* cells and different concentrations of amino acids were added to the sediment prior to the addition of extraction buffer and heating of the samples. After cell disruption by either ultra-sonication or bead-beating, proteins were separated from the sediment particles by centrifugation or in the BioRad Prep Cell. After TCA precipitation, protein extracts were processed by SDS-PAGE or FASP. The optimized extraction workflow was carried out on 10 g of sediment supplemented with amino acids. After cell disruption via ultra-sonication in urea/thiourea, proteins were extracted from the sediment using the prep cell. Finally, all samples were desalted using C18 ZipTips and measured by LC–MS/MS.

For optimization of the protein extraction approach, proteins were extracted in triplicates with different workflows (Fig. 1) from 5 g of sediment (wet weight) as starting material. All steps were performed in protein LoBind® tubes (Eppendorf, Hamburg, Germany).

For evaluation of the protein extraction efficiency, first, the optimal amount of *E. coli* cells was estimated, that allowed for sufficient protein recovery from the sediments without suppressing the signal of sediment-derived peptides. For this purpose, the sediment material was combined and thoroughly mixed with 1 ml of an *E. coli* cell suspension with increasing cell numbers (approximately 8 × 10^6^, 8 × 10^7^, 8 × 10^8^, or 8 × 10^9^ cells) for 30 min on ice and mixed with 5 ml urea/thiourea buffer (6 M urea/3 M thiourea). Samples were heated for 15 min at 100°C and treated with ultra-sonication (pulse sonication, 3 × 60 s, 70% intensity) for cell disruption. After centrifugation (10 000 × g, 10°C, 15 min), the supernatant was transferred into a new tube and proteins were precipitated using trichloroacetic acid (TCA, final concentration of 25% w/v) at 4°C overnight. After centrifugation (12 000 × g, 10°C, 15 min), the protein pellet was washed twice with ice-cold 80% (v/v) acetone, once with ice-cold 100% acetone, and air-dried, subsequently. Protein pellets were resuspended in 30 μl 2× Laemmli sample buffer (125 mM Tris–HCl pH 6.8, 20% (v/v) glycerol, 4% (w/v) SDS, 3.75% (v/v) β-mercaptoethanol, 100 mM dithiothreitol (DTT), 0.04% (w/v) bromophenol blue). After heating at 98°C for 5 min, protein extracts were loaded onto an SDS-gel, and electrophoresis was run at 180 V for approximately 10 min until the ion front had moved about 1.5 cm into the gel. The gel was fixed with 10% (v/v) acetic acid in 40% (v/v) ethanol for 15 min and stained with 80% (v/v) colloidal Coomassie G-250 solution (10% (w/v) ammonium sulfate, 1.2% (v/v) phosphoric acid, 0.1% (w/v) colloidal Coomassie G-250) in 20% (v/v) methanol overnight [36]. Gel pieces containing proteins were excised and cut into small 1 mm sized cubes [37]. The Coomassie staining was removed by washing the gel pieces in a washing solution (200 mM ammonium bicarbonate, 30% (v/v) acetonitrile) three times for 15 min at 37°C and 900 rpm. Subsequently, gel pieces were dried in a vacuum centrifuge and proteins were in-gel digested with 0.1 μg trypsin (Promega, Madison, Wisconsin, USA) for 14 h at 37°C. Peptides were eluted from the gel pieces in an ultra-sonication bath and desalted using ZipTip cleanup according to the manufacturer’s protocol before LC–MS/MS measurement. To evaluate the recovery of *E. coli* proteins from the sediment, the whole procedure was repeated for the pure cell suspensions without sediment material.

Second, the capacity of amino acids to block potential protein binding sites in the sediment material was tested. After the addition and incubation of *E. coli* cell suspension (8 × 10^8^ cells) with the sediment material, samples were mixed with 2 ml of distilled water as a control or an amino acid mixture of the polar positive amino acids arginine, histidine, and lysine (ratio 1:1:1, pH 7) [17] to a final concentration of 25% (w/v) or 50% (w/v) of amino acids. *E. coli* containing sediment samples were incubated with the amino acids for 30 min on ice and processed as described above.

Third, for testing of different cell disruption approaches, sediment samples prepared with 8 × 10^8^  *E. coli* cells and amino acids (50% (w/v)) were heated for 15 min at 100°C in a thermo-shaker without shaking and treated with ultra-sonication or bead-beating. The ultra-sonication procedure was applied at 97% intensity. About 2 ml of glass beads (0.25–0.5 mm) were added for the bead-beating process to the prepared sediment samples which were shaken four times with 5.5 m/s for 40 s with 5 min breaks on ice in between the cycles. After cell disruption, samples were centrifuged and processed as described above.

Alternatively to SDS-PAGE, the filter-aided sample preparation (FASP) protocol was tested as described by Wiśniewski [38] using protein pellets obtained as described above (8 × 10^8^  *E. coli* cells, 50% (w/v) amino acids, ultra-sonication (97% intensity)). Proteins on the filter were reduced with 100 μl of 100 mM Tris(2-carboxyethyl)phosphine hydrochloride (15 min, 65°C) and alkylated with 100 μl 100 mM Iodoacetamide (20 min in the dark at RT). Afterward, proteins were digested for 14 h at 37°C using 0.5 μg trypsin (Promega). Peptides were eluted from the filter with 0.05 M ammonium bicarbonate and desalted using ZipTip cleanup according to the manufacturer’s protocol before LC–MS/MS measurement.

Finally, as a comparison to the previously described procedures including a centrifugation step to separate proteins from the sediment, the sediment-buffer mixture was directly mixed with 5 ml 5x Laemmli sample buffer and heated at 98°C for 10 min after cell disruption via ultra-sonication (97% intensity). Following, the whole mixture was loaded onto a self-made gel (20 ml 12% Acrylamide/Bis (Rotiphorese® Gel 30, Carl-Roth, Karlsruhe, Germany) as separating gel overlaid with 15 ml 4% Acrylamide/Bis as stacking gel) in a Model 491 Prep Cell from BioRad (Hercules, California, USA) modified after Moore *et al.* [31]. Electrophoresis was performed at a constant power of 40 W. Proteins were automatically eluted from the gel in the running buffer (3% (w/v) Tris, 14.4% (w/v) glycine, 1% (w/v) SDS, pH 8.3) after reaching the end of the gel. With a sample collector, every hour a new fraction of eluted proteins was collected (approximately 6 ml per fraction). The ion front was eluted after approximately four hours of continuous electrophoresis. Therefore, the first three eluted fractions were discarded. Afterward, proteins were precipitated using TCA precipitation, and resulting protein pellets were processed by an additional SDS-PAGE, in-gel digestion, and peptide cleanup as described above.

### Final protein extraction protocol using electro-elution

Proteins were extracted from 10 g sediment (wet weight) after mixing with 4 ml of a 50% (w/v) amino acid solution and incubation for 30 min on ice. Subsequently, 10 ml of urea/thiourea buffer was added before heating for 15 min at 100°C and three times ultra-sonication for 60 s using pulse sonication at 97% intensity. The sediment-buffer mixture was combined with 5 ml 5x Laemmli sample buffer and heated again for 15 min at 100°C. Afterward, the whole sediment slurry was loaded onto a self-cast gel in the Prep Cell (15 ml 12% separating gel, 15 ml 4% stacking gel). Electrophoresis was performed for 14 h at constant power (30 W). Proteins were eluted from the gel in a running buffer and collected in fractions of one hour each. After completing the electrophoresis, proteins were TCA precipitated, and resulting protein pellets were resuspended and processed by SDS-PAGE and in-gel digestion before desalting with ZipTips.

### Liquid chromatography tandem mass spectrometry measurements

Peptides were suspended in 10 μl 0.1% (v/v) acetic acid in water and separated by reversed-phase chromatography using an EASY nLC 1200 (Thermo Fisher Scientific, Waltham, USA) with self-packed columns (outer diameter 360 μm, inner diameter 100 μm, length 20 cm) filled with 3 μm diameter C18 particles (Dr. Maisch, Ammerbuch-Entringen, Germany) in a one-column setup at a constant temperature of 45°C. Peptides were separated by applying a binary non-linear gradient from 0–95% (v/v) acetonitrile in 0.1% (v/v) acetic acid over 100 min. The LC was coupled online to an LTQ Orbitrap Elite mass spectrometer (Thermo Fisher Scientific, Waltham, USA) with a spray voltage of 2.5 kV. After a survey scan in the Orbitrap (r = 60 000), MS/MS data were recorded for the twenty most intense precursor ions in the linear ion trap. Singly charged ions were not considered for MS/MS analysis. The lock mass option was enabled throughout all analyses.

### Peptide and protein identification

For database search, metagenomic sequences obtained as described in the supporting information for eight sediment cores sampled in the same area as the sediment core analyzed at the proteomic level were combined and filtered for redundant sequences [39]. In addition, entries with more than 97% sequence similarity were removed using CDhit [40]. Common laboratory contaminants were included in the database, as well as the proteome of *E. coli* K12 as standard during the optimization of the protein extraction approach. The resulting database contained approximately 1.4 million entries. Reversed sequences were searched to determine the false discovery rate.

After mass spectrometric measurement, a database search was performed on Mascot (v. 2.7.0.1) [41] using the metagenome-based database (including *E. coli* proteins during the optimization process). No missed cleavages were allowed, and an oxidation of methionine was considered a variable modification for all searches. For samples prepared by FASP protocol, carbamidomethylation of cysteine was considered as a fixed modification, additionally. Search results were further processed in Scaffold (v. 5.2.2) [42], performing an additional X!Tandem (v. 2017.2.1.2) [43] search. Peptide identifications were accepted if they could be determined with a probability of more than 95.0% by the Peptide Prophet algorithm [44] with Scaffold delta-mass correction. Protein identifications were accepted if they could be detected with a probability of more than 99.0% and contained at least 2 identified peptides. Protein probabilities were assigned by the Protein Prophet algorithm [45]. Proteins that contained similar peptides and could not be distinguished based on MS/MS analysis alone were grouped to satisfy the principles of parsimony. The quantification of protein groups was based on the quantitative value “normalized weighted spectra” determined in Scaffold. Functional annotation of proteins was performed using eggnog-mapper v2 [46] and data visualization was performed in RStudio (Posit).

## Results

### Supplementing *Escherichia coli* cells as an internal standard

**Figure 2:**
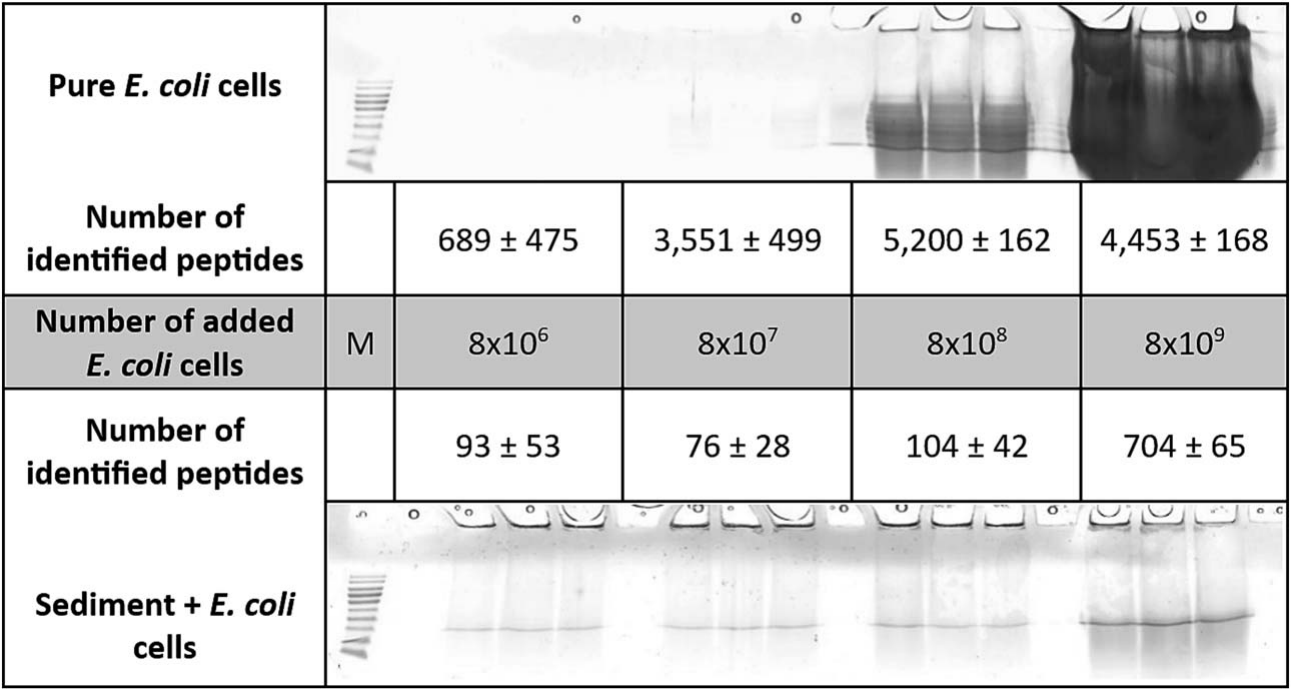
SDS-gels and number of identified peptides obtained from different numbers of *E. coli* cells (top) or *E. coli* cells spiked in 5 g of sediment (bottom). Experiments were performed in triplicates, means and standard deviations of identification numbers within replicates were calculated. Samples were mixed with urea/thiourea and cells were disrupted by heating and ultra-sonication. Sediment particles were removed by centrifugation and proteins were enriched by TCA precipitation before SDS-PAGE. Peptide identification was performed as described. M = marker.

In order to evaluate the effectiveness of the different protein extraction methods, an internal standard of *E. coli* cells was added to the sediment material prior to cell disruption. SDS-PAGE was used to process protein extracts from different numbers of *E. coli* cells from pure cultures (Fig. 2 top) and from *E. coli* cells added to 5 g of sediment (Fig. 2 bottom). In the SDS-gel, a clear correlation was observed between the number of pure *E. coli* cells used and the signal intensities. As expected, the number of extracted proteins increased with higher cell numbers. In contrast, such a strong correlation could not be observed after the addition of different numbers of *E. coli* cells to sediment material. Even with a smaller visual effect on the SDS-gel, the number of *E. coli* proteins and peptides identified from the sediment increased significantly in the samples supplemented with a higher number of *E. coli* cells (Fig. 2). However, the number of identified *E. coli* peptides extracted from the sediment remained lower compared to the number of identified peptides extracted from pure *E. coli* cells. On average, about 700 unique peptides and 146 proteins from *E. coli* were identified from sediment samples enriched with the highest number of cells, whereas the same number of peptides were identified using the lowest number of pure cells from *E. coli*. More than 5 000 peptides corresponding to about 800 proteins were identified using 8 × 10^8^ pure *E. coli* cells, while only 104 peptides could be recovered from the sediment supplemented with the same amount of *E. coli* cells.

### Pretreatment of sediment and protein extract processing

**Figure 3:**
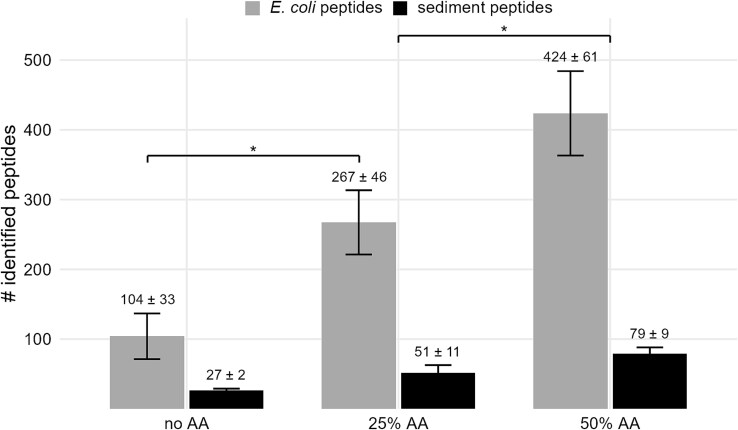
Peptides identified in sediment samples treated with different concentrations of an amino acid solution. The experiments were carried out in triplicates, means and standard deviations of identification numbers within replicates were calculated. About 8 × 10^8^  *E. coli* cells were added to the sediment samples. After the addition of positive polar amino acids solution (25% or 50% AA) or water (no AA), samples were mixed with urea/thiourea, and cells were disrupted by heating and ultra-sonication as described.

Only a minimal improvement in protein extraction efficiency was observed on protein and peptide level when using a 10% amino acid solution compared to an untreated control sample (Fig. S1). However, the number of identified peptides doubled when the sediment was mixed with a 25% amino acid solution compared to the untreated control (Fig. 3). Furthermore, the number of identified peptides derived from the sediment material increased from 27 to about 50 peptides, corresponding to 10 to 20 proteins, respectively. The positive effect was further enhanced when a 50% amino acid solution was used, resulting in four times more identified *E. coli* peptides and three times more sediment peptides compared to the untreated control sample.

**Figure 4:**
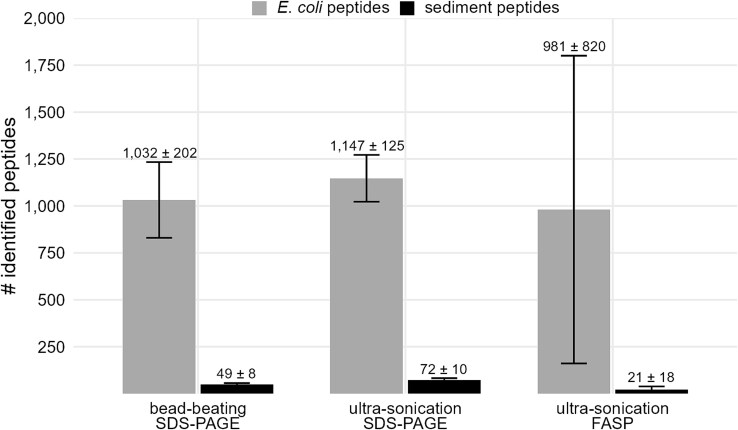
Peptides identified according to different strategies for cell disruption and protein processing. The experiments were carried out in triplicates, means and standard deviations of identification numbers within replicates were calculated. Sediment samples were spiked with approximately 8 × 10^8^  *E. coli* cells. After addition of positive polar amino acids solution (50%), samples were mixed with urea/thiourea. Cells were disrupted with bead-beating or ultra-sonication.

For the following approaches, the sediment samples were mixed with *E. coli* cells and a 50% amino acid solution. The sediment samples were then heated and cells were disrupted either by ultra-sonication or bead-beating before extracted proteins were further processed by TCA precipitation and SDS-PAGE. The increased intensity of ultra-sonication from 70% (Fig. 3) to 97% (Fig. 4) leads to a 3-fold increase in *E. coli* peptide identifications, and a doubling of protein identifications compared to the previous experiments using the 50% amino acid solution. In contrast, similar numbers of *E. coli* peptides and proteins were identified by ultrasonic treatment at an intensity of 97% and bead-beating (Fig. 4). The reproducibility between the replicates was higher after ultra-sonication treatment for cell disruption compared to bead-beating (Fig. S2). The number of identified sediment peptides was higher for ultra-sonicated samples compared to bead-beaten samples. In addition to the SDS-gel approach, proteins extracted from ultra-sonicated cells were processed directly via FASP. The average number of identified *E. coli* peptides and proteins was similar to the aforementioned approaches, but the standard deviation was very high.

### Protein extraction through electrophoresis using Prep Cell

**Figure 5:**
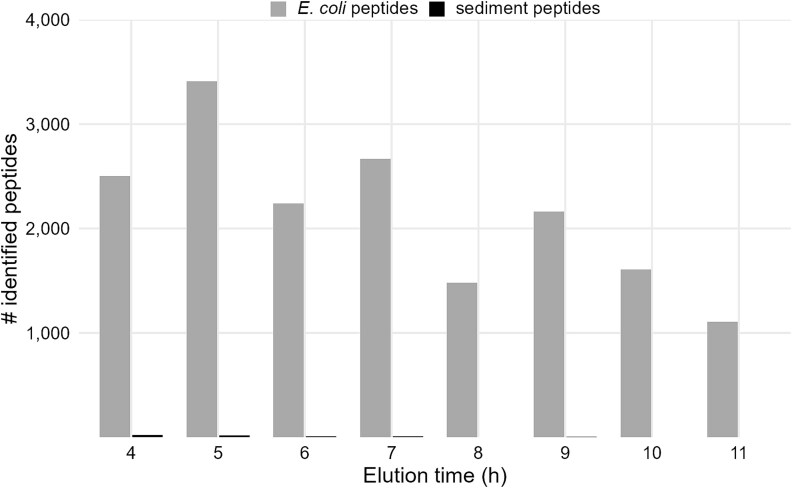
Peptides identified in eluted fractions of the electro-elution approach. The sediment sample was spiked with approximately 8 × 10^8^  *E. coli* cells. After the addition of positive polar amino acids solution, the sample was mixed with urea/thiourea, and cells were disrupted by heating and ultra-sonication as described. Proteins were electro-eluted from the resulting sediment slurry using the BioRad Prep Cell. After ~4 h, the ion front and the first proteins were eluted.

Although the addition of amino acids could increase the number of identified peptides and proteins, less than 30% of *E. coli* proteins were identified from the sediment (Table S1). To further reduce the loss of all sediment-bound proteins during centrifugation steps, the so-called “slurry” approach [31] was tested. By applying an electric field directly to the sediment-buffer mixture, proteins are electro-eluted from the sediment and pulled into an SDS-gel. Fractionated proteins were eluted from the tube gel in fractions of 1 h each with a flow rate of approximately 6 ml per hour. Proteins were precipitated with TCA and further processed by SDS-PAGE, in-gel digestion and ZipTip cleanup before MS/MS analysis. More than 5 900 *E. coli* peptides, corresponding to 982 proteins were identified, with up to 3 400 peptides (600 proteins) being identified in single elution fractions using the slurry extraction in the Prep Cell (Fig. 5). Although the number of identified *E. coli* proteins was quite high, almost no sediment-derived proteins were identified with the metagenome-based database.

### Applying the optimized workflow to Barents Sea sediment

**Figure 6:**
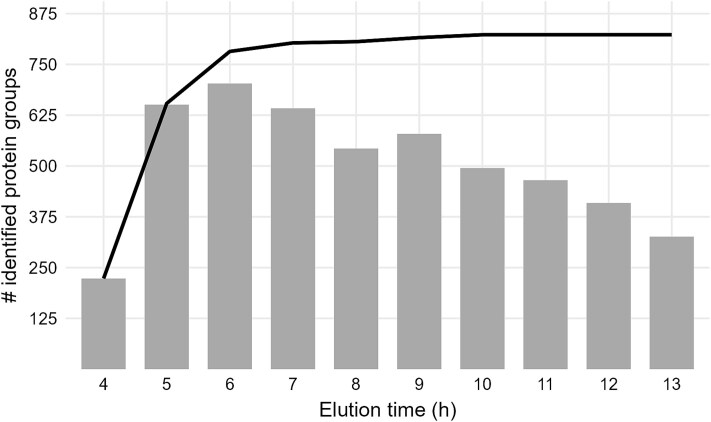
Number of identified protein groups in eluted fractions. After the addition of positive polar amino acids solution to the sediment, the sample was mixed with urea/thiourea, and cells were disrupted by heating and ultra-sonication. Proteins were electro-eluted from the resulting sediment slurry in the BioRad Prep Cell. Grey bars indicate the number of proteins groups identified in the respective fraction. The black line shows accumulated numbers of proteins groups.

Finally, the optimized workflow, including the addition of amino acid solution prior to cell lysis via ultrasonic treatment and electro-elution of the proteins, was applied to 10 g of Barents Sea sediment without internal *E. coli* cells as standard. In the 5 h after elution of the ion front (5–9 h), about 500 to 700 protein groups were identified in the respective fractions (Fig. 6). The number of identified protein groups decreased in later fractions to 300 protein groups. A total of 823 protein groups (6 179 unique peptides) were identified, most of which were found in the first three fractions, including the ion front fraction (4–6 h of the electrophoresis). No additional unique protein groups were identified in fractions eluted after 10 h of electrophoresis.

**Figure 7:**
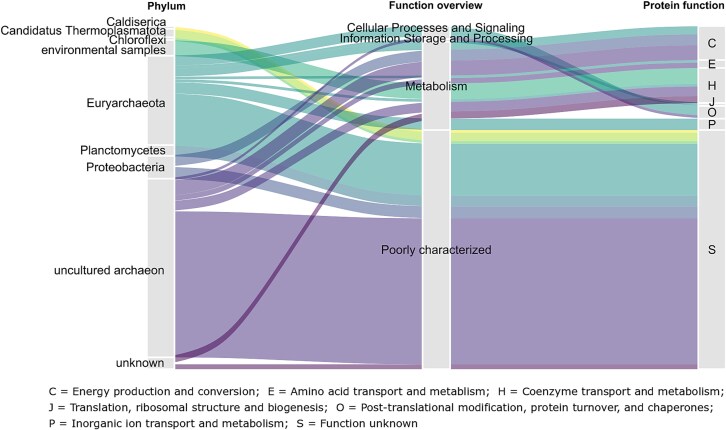
Proportion of assigned phyla and respective functions of identified protein groups. Phyla were determined based on the metagenomic information. Protein functions were determined by EggNOG based on COG categories (Table S2) [46].

The majority of the identified protein groups were assigned to uncultured archaea (48%) and Euryarchaeota (24%). Other abundant taxa included Proteobacteria (8%), and Planctomycetes (3%) (Fig. 7). Proteins identified for the first time in environmental samples made up approximately 4% of all proteins, whereas about 6% of the proteins belonged to unknown phyla. While 94% of the proteins in this environment could be assigned to a known phylum, one third of them could not be classified into a known class, indicating a high proportion of uncharacterized organisms. The functions of the identified protein groups were predicted by EggNOG [46] based on COG categories. About 63% of the proteins were poorly characterized. The majority of proteins with known functions are involved in energy production and conversion (9%) or coenzyme transport and metabolism (9%).

## Discussion

Microorganisms in marine sediments play a significant role in the biogeochemical cycling of carbon, nitrogen and sulfur, as well as in the potential bioremediation of petroleum hydrocarbons and heavy metals [47, 48]. Several metagenomic studies have been conducted to reveal the microbial diversity and metabolic potential in marine sediments [49–51]. However, metaproteomic approaches can provide complementary information by identifying the metabolic pathways that are actively used and the microorganisms that contribute to these processes [25]. In metaproteomic analyses, the effectiveness of protein extraction from the sample material is crucial. A high clay content in the sediment material reduces the efficiency of protein extractions [10, 32] due to the binding process between proteins and the clay or sediment particles [16]. We were able to confirm this effect by comparing the number of identified peptides and proteins extracted from either pure *E. coli* cell cultures or from cells spiked into the sediment. Using different numbers of *E. coli* cells from the pure cell suspension, the number of identified peptides and proteins increased in correlation with increasing cell numbers and protein amount. In protein extracts from 8 × 10^9^ cells, the signal of less abundant peptides was suppressed during MS measurement due to the overload of highly abundant peptides since proteins were not fractionated, leading to a reduced number of identifications compared to the lower amount of cells (8 × 10^9^) [52]. In contrast, only a small increase in the number of proteins extracted was observed with increasing cell number when the cells were added to the sediment material. Most *E. coli* proteins could not be extracted from the sediment.

Nicora and colleagues increased the recovery of *E. coli* proteins from sediment with low clay content (approximately 5% clay) from 8% to 63% by mixing a 10% solution of polar positive amino acids into the sediment before cell lysis [17]. However, applying the same treatment to our samples did not result in a notable increase in the number of identified peptides and proteins. Since the low concentration of amino acids as proposed by Nicora and colleagues was not sufficient for effective protein extraction from clay-rich sediments, we increased the concentration of amino acids to 25% and 50%, and observed a positive effect on the efficiency of protein extraction, with recoveries of 8% and 13%, respectively.

Increasing the intensity of the ultra-sonication treatment for cell disruption from 70% to 97% led to a higher number of extracted proteins. There was no significant difference in efficiency between ultra-sonication and bead-beating for cell disruption in terms of *E. coli* peptide identification numbers, but ultrasonic treatment at 97% intensity was superior for the extraction of sediment-derived proteins. In addition, on average, similar numbers of *E. coli* peptides were identified by SDS-PAGE and in-gel digestion compared to the FASP protocol and filter digestion. While previous studies have shown that FASP is superior to SDS-PAGE and in-gel digestion [53], we found that FASP was not suitable for our samples. The protein extracts remained dirty, possibly due to co-extracted humic substances, resulting in clogging of the filter surface and subsequently in a high variance of peptide and protein identifications between replicates. Similar problems occurred during preliminary tests using the SP3 protocol [54] for sample preparation. Since protein extracts remained dirty, protein binding and purification on magnetic SP3 beads was inefficient and yielded very low peptide and protein identifications (data not shown).

Despite the amino acid treatment, the recovery of *E. coli* peptides remained low (about 22%) after removing sediment particles by centrifugation. This indicates that a significant proportion of the released proteins became bound to sediment particles and was lost during the centrifugation step [15]. To overcome this problem, we tested a modified version of the so-called ‘slurry’ extraction method proposed by Moore and colleagues [31], which does not require centrifugation and allows for an improved recovery of proteins from the sediment. Proteins are extracted from the sediment and pulled into an SDS-gel by electrophoresis. In a previous study, we used a similar technique utilizing electro-elution by SDS-PAGE to recover proteins enriched on affinity beads [37]. In this study, we have already shown that electrophoresis is a very efficient method for eluting proteins that are firmly bound to a high-affinity matrix. Moore and colleagues used either flat 1D gels or a tube gel in the Mini Prep Cell from BioRad. In both approaches, electrophoresis was run until the ion front moved a few centimeters into the gel and protein-containing gel pieces were excised. Often, problems occurred by charged sediment particles, which were also affected by the electric field and resulted in physically tearing of the gel surface [31, 34, 55]. To reduce the impact of this effect, we used the model 491 Prep Cell from BioRad, which allows protein elution from the gel during separation. Although we also observed the tearing effect with the Prep Cell, it did not cause any further problems because the proteins were eluted directly from the gel and the remaining gel matrix was discarded after electrophoresis. The Prep Cell allowed for loading of a larger sample volume compared to flat 1D gels used by Moore and colleagues [31, 34], resulting in a higher protein yield. Using this technique, we were able to identify 14% more *E. coli* peptides from the prepared sediment compared to the pure cell suspension, which was processed using the non-optimized workflow. The observed increase in peptide identifications may be partly attributed to the pre-fractionation of proteins during the “slurry” extraction procedure [7]. However, our optimized workflow enables efficient recovery of proteins from the clay-rich sediment. Previous studies have reported recovery rates of supplemented proteins ranging from 63% [17] to 85% [32]. Despite the high number of identified *E. coli* proteins, the number of identified sediment-derived protein groups was rather low. Most likely, the high abundance of *E. coli* proteins and corresponding peptides suppressed the signal of less abundant sediment-derived peptides during the MS analysis [52]. Furthermore, the high complexity in environmental samples like marine sediments can reduce the number of protein identifications. Since the *E. coli* cells were not allowed to grow and establish in the sediment, we cannot preclude the possibility that cell disruption and therefore protein extraction is more sufficient on loosely bound cells compared to endogenous cells. However, it was important for us to use an internal standard to be able to compare the efficiency of protein extraction between the individual experiments. The overall number of identified *E. coli* peptides and proteins was limited compared to other studies, most likely due to the missing of a protein fractionation step and the limited measuring depth of the used LTQ-Orbitrap system.

The optimized protein extraction workflow was applied to marine sediment from the Barents Sea, resulting in the identification of 823 sediment-derived protein groups. This number is higher than that of most other metaproteomic analyses of marine sediments. For instance, Moore and colleagues identified between 11 and 130 proteins using electrophoresis for protein extraction [31, 34, 55], while other studies identified between 250 and 650 proteins from marine sediments using a Nycodenz gradient centrifugation, the Tri-reagent, or simple centrifugation after cell disruption for protein identification [24, 27, 56]. When using continuous electrophoresis for protein extraction, most of the identified proteins appear in the first fractions after the ion front. The number of protein identifications in later fractions is still relatively high, but most of the proteins could already be found in the previous fractions. For future studies, it may be beneficial to use a higher volume of the gel matrix and lower power during the electrophoresis to achieve better fractionation and thus improve protein identification. Despite suboptimal separation of proteins in the gel, the extraction efficiency was very good.

The microbial community in the sediment was dominated by archaea and Euryarchaeota. In other studies, Thaumarchaeota have been found to represent the largest proportion of archaea in marine sediments, while Euryarchaeota were also present in a noteworthy portion (up to 23% of the archaeal community) [57–59]. As previously observed in sediments from the Barents Sea [60] and other marine sediments [61–63], Proteobacteria, Planctomycetes, and Chloroflexi were the most prominent bacterial phyla. Interestingly, a majority of identified proteins were assigned to archaea rather than bacteria, although other studies found that bacteria clearly dominate over archaea in subseafloor sediment [1]. However, most of the identified proteins are poorly characterized. Nevertheless, proteins involved in metabolism, cellular processes, and signaling, and information storage and processing were also identified.

In conclusion, several steps of sample preparation and protein extraction had to be rationally combined and adapted for effective protein extraction from clay-rich sediments. The concentration of amino acids to block protein binding sites on sediment particles [17] had to be substantially increased for this type of sediment. Furthermore, previous techniques for electro-elution of proteins were mainly based on flat 1D SDS-gels [31, 34, 37], which only allow for very limited sample volumes. For sediment samples with low biomass, the applicable sample volume (<1 ml) is insufficient for sufficient protein yield. The BioRad Prep Cell 491 allowed for a larger sample volume and direct elution of proteins from the gel matrix, thus eliminating the negative effect of gel-tearing. The method shown here can be used for effective protein extraction, even from clay-rich sediment with low biomass.

## Supplementary Material

Supporting_information_ycaf074

Supporting_information_identified_proteins_ycaf074

## Data Availability

Proteome mass spectral data are available in the ProteomeXchange Consortium via the PRIDE partner [64] repository with the identifier (PXD054260). All other data are available within the article and supporting information files.
