# Supplementary material for: Systematic evaluation of protein extraction for metaproteomic analysis of marine sediment with high clay content
Source: ISME Commun. 2025 May 22;5(1):ycaf074. doi: 10.1093/ismeco/ycaf074 (PMC12192440; doi:10.1093/ismeco/ycaf074)
Supplement: Supporting_information_ycaf074 [file supporting_information_ycaf074.pdf]

Supporting Information to

Systematic evaluation of protein extraction for metaproteomic analysis of marine sediment with high clay content

Anne Ostrzinski[1], Benoît J. Kunath[2], André Rodrigues Soares[3], Cedric C. Laczny[3], Rashi Halder[3], Jens Kallmeyer[4], Rolando di Primio[5], Paul Wilmes[3,6], Alexander J. Probst[2], Anke Trautwein-Schult[1], Dörte Becher[1]

[1] Department of Microbial Proteomics, Institute of Microbiology, University of Greifswald, Germany

[2] Luxembourg Centre for Systems Biomedicine, University of Luxembourg, Esch-sur-Alzette, Luxembourg

[3] Department of Environmental Metagenomics, Research Center One Health Ruhr, University Alliance Ruhr, Faculty of Chemistry, University of Duisburg-Essen, Essen, Germany

[4] Section Geomicrobiology, GFZ Helmholtz Centre for Geoscience, Potsdam, Germany

[5] Exploration Manager Play Analysis & Access, Aker BP, Norway

[6] Department of Life Sciences and Medicine, Faculty of Science, Technology and Medicine, University of Luxembourg, Esch-sur-Alzette, Luxembourg

DNA extraction and metagenome creation

Samples were subjected to DNA extraction using the DNEasy PowerMax Soil Kit (Qiagen, Hilden, Germany). Due to the complexity of sample material, the following adjustments had to be made to the manufacturer's instructions. For 2.5 g of starting sediment material, the final elution was performed in two steps using two times 2.5 ml of solution C6, two incubations, and two centrifugation steps. Eluted DNA was then concentrated by adding 510 µl of a 3 M sodium acetate (pH 5.2)/glycogen (0.4 µg/µl) solution and incubated for one hour at -20 °C and then centrifuged to pellet DNA. After ethanol cleaning

and air-drying, DNA was resuspended in 100 µl of 10 mM Tris buffer. Finally, DNA was cleaned and concentrated using the Zymo DNA Clean & Concentrator (Zymo, Irvine, California, USA) kits following the manufacturer's instructions and with a final elution of 35 µl.

For metagenomic library preparation using the QIAseq FX DNA library UDI A/B kit (Qiagen), 32.5 µl of purified and concentrated DNA were used irrespective of the individual sample concentrations. The genomic DNA was enzymatically fragmented for 10 min and DNA libraries were prepared with 7 PCR cycles for the PCR-less samples, and without extra PCR steps for the PCR-free samples. The average library sizes were ±400 bp. Prepared libraries were quantified using a Qubit fluorometer (Thermo Fischer, Waltham, Massachusetts, USA) and quality-checked on a Bioanalyzer (Agilent, Santa Clara, California, USA). Sequencing was performed on an Illumina NextSeq2000 instrument using 2 × 151 bp read length at the Luxembourg Centre for Systems Biomedicine (LCSB) Genomics Platform, aiming at an average of 10 Giga base pairs (Gbps) per sequencing library. Raw Illumina short reads were trimmed and quality checked using bbduk and sickle (1). The reads passing quality-control were then assembled using metaviralSPAdes (2) (k-mers 21,33,55,77,99), generating scaffolds for the viral fraction of the metagenome. Reads mapping to viral scaffolds were then removed from the input quality-controlled reads and used as input to metaSPAdes (3), which assembled the prokaryotic fraction of the metagenome. Scaffolds smaller than 1,000 bp were removed and genes were predicted using prodigal (4). Annotation of predicted gene amino acid sequences was conducted with diamond (5) using FunTaxDB (6) considering a minimum p-value of 10^{-5}. Annotation strings were added to the headers of amino acid sequences using a dedicated awk script to permit their use as a database for protein identification.

Pre-test for amino acid supplementation

In a first experiment, the pretreatment of sediments with amino acids was tested with solutions of 10% (as proposed by Nicora *et. al*) (7) or 50% polar positive amino acids (Fig. S1). For this experiment, approx. $8x10^9$ *E. coli* cells were added to the sediment before amino acid treatment and cell disruption. The

number of identifications increased for *E. coli* proteins and peptides but decreased for sediment-proteins using the 50% amino acid solution. We assumed, that the high concentration of *E. coli* proteins compared to sediment-proteins, lead to a suppressed signal of sediment-proteins during MS measurement and decided to reduce the number of amended *E. coli* cells to approx. $8x10^8$ cells for the later experiments.

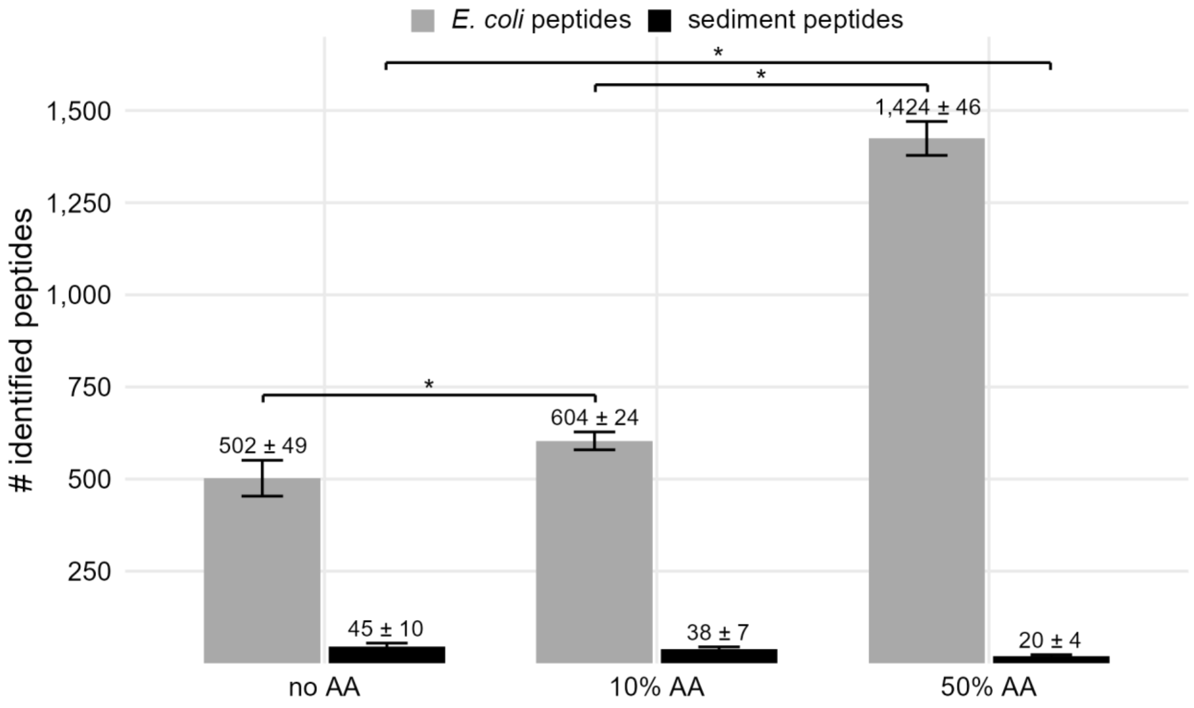

Fig S1: Identified peptides in sediment samples treated with different concentrations of an amino acid solution. Experiments were performed in triplicates, means and standard deviations of identification numbers within replicates were calculated. Sediment samples were spiked with approx. $8x10^9$ *E. coli* cells. After the addition of positive polar amino acids solution (10% or 50% AA) or water (no AA), samples were mixed with urea/thiourea, and cells were disrupted by heating and ultra-sonication (70% intensity).

Comparing different techniques for cell disruption and sample processing

In triplicates, sediments (amended with approx. $8x10^8$ *E. coli* cells) were processed by bead-beating and ultra-sonication (97% intensity) prior to centrifugation for removal of sediment particles, and SDS-PAGE of the protein extracts. Additionally, one batch of ultra-sonicated samples was processed using FASP instead of SDS-PAGE. The overlap of identified *E. coli* proteins between replicates was used as a measure for reproducibility (Fig. S2). In samples processed by bead-beating and SDS-PAGE, 72.1% of proteins and 67% of peptides were identified in at least two of the three replicates. With 81.9% of proteins and 71% of peptides identified in at least two of three replicates, the reproducibility was higher in samples processed

by ultra-sonication and SDS-PAGE. The reproducibility of FASP samples was lower, 66.8% of proteins and 55% of peptides were identified in two of three replicates, but in one of the replicates only very few proteins (15 proteins) could be identified at all.

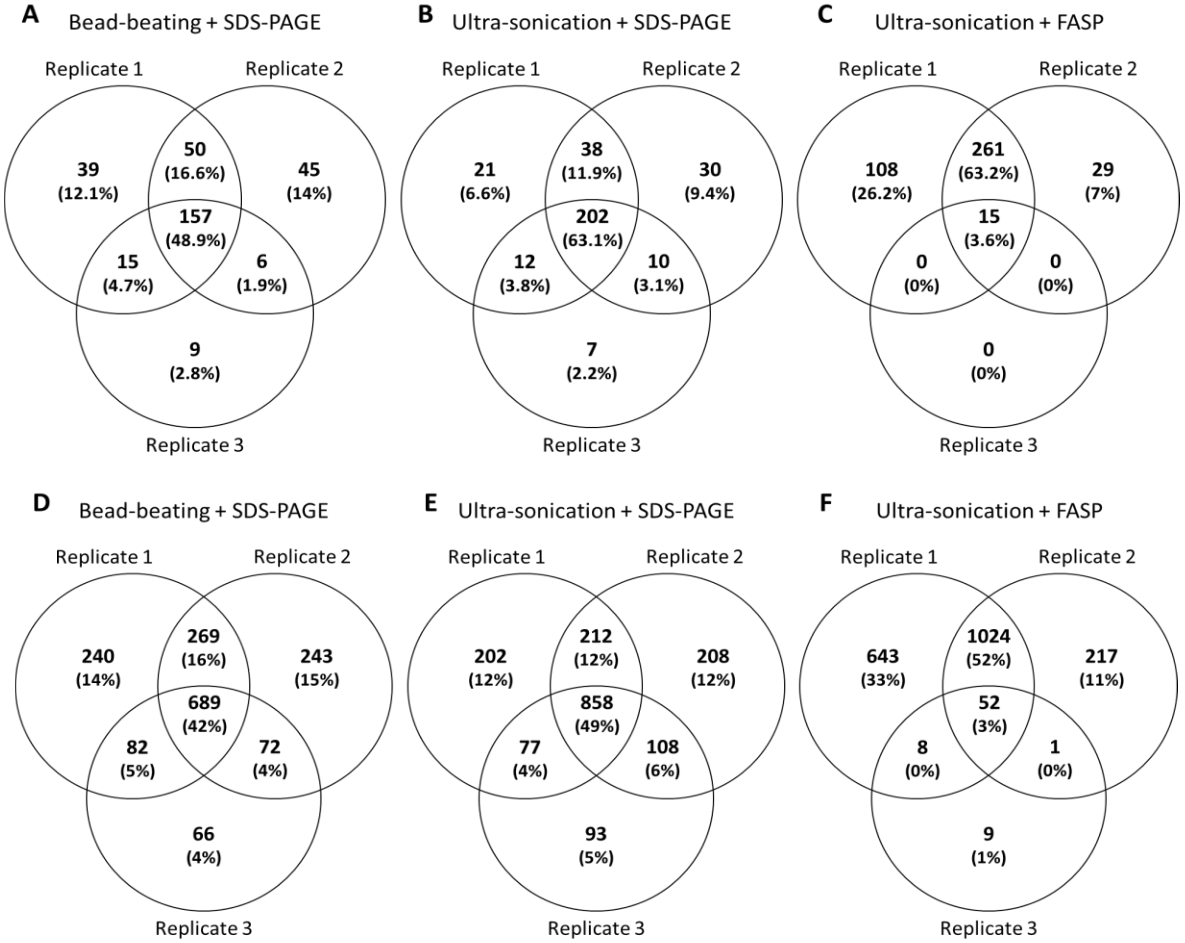

Fig S2: Overlap of identified *E. coli* proteins (A-C) and peptides (D-F) between replicates after different cell disruption and protein processing strategies. Sediment samples were spiked with approx. $8x10^8$ *E. coli* cells. After addition of positive polar amino acids solution (50%), samples were mixed with urea/thiourea. Cells were disrupted with bead-beating (A and D) or ultra-sonication (B, C, E, and F). Sediment particles were removed by centrifugation and proteins were enriched by TCA precipitation prior to SDS-PAGE, in-gel digestion (A, B, D, and E) or FASP (C and F).

Overview of recovery rates of *E. coli* proteins across different extraction experiments

E. coli cells have been added to the sediments prior to cell disruption. The numbers of *E. coli* proteins and peptides identified from sediment samples were compared to protein and peptide identification numbers from a pure *E. coli* culture, and were used as a measure of extraction efficiency (Table S1).

Table S1: Number of identified *E. coli* peptides and proteins from differently treated sediment samples. Experiments were performed in triplicates, means of identification numbers within replicates were calculated. MS data were searched with Mascot against a metagenome-based database containing the *E. coli* K12 proteome. Search results were further reanalyzed in Scaffold using X!Tandem.

Sample	Unique Peptides	Proteins	Protein recovery from sediment (%)
E. coli only	5,194	819	-
No AA	104	20	2
25% AA	267	64	8
50% AA	424	106	13
Bead-beating + SDS-PAGE	1032	216	26
US + SDS-PAGE	1,147	232	28
US + FASP	980	225	27
50% AA + ‘Slurry’	5,910	980	120

Table S2 containing all information about identified proteins in the core samples is provided as a separate file.

References

1. Bushnell B. BBMap: A Fast, Accurate, Splice-Aware Aligner.: Lawrence Berkeley National Lab. (LBNL), Berkeley, CA; 2014. Available from: URL: https://github.com/najoshi/sickle.

2. Antipov D, Raiko M, Lapidus A, Pevzner PA. Metaviral SPAdes: assembly of viruses from metagenomic data. Bioinformatics 2020; 36(14):4126–9.

3. Nurk S, Meleshko D, Korobeynikov A, Pevzner PA. metaSPAdes: a new versatile metagenomic assembler. Genome Res 2017; 27(5):824–34.

4. Hyatt D, Chen G-L, Locascio PF, Land ML, Larimer FW, Hauser LJ. Prodigal: prokaryotic gene recognition and translation initiation site identification. BMC Bioinformatics 2010; 11:119.

5. Buchfink B, Reuter K, Drost H-G. Sensitive protein alignments at tree-of-life scale using DIAMOND. Nat Methods 2021; 18(4):366–8.

6. Bornemann TLV, Esser SP, Stach TL, Burg T, Probst AJ. FunTaxDB database for uBin software; 2022.

7. Nicora CD, Anderson BJ, Callister SJ, Norbeck AD, Purvine SO, Jansson JK et al. Amino acid treatment enhances protein recovery from sediment and soils for metaproteomic studies. Proteomics 2013; 13(18-19):2776–85.
